# Supplementary material for: The photocycle of orange carotenoid protein conceals distinct intermediates and asynchronous changes in the carotenoid and protein components
Source: Sci Rep. 2017 Nov 14;7:15548. doi: 10.1038/s41598-017-15520-4 (PMC5686206; doi:10.1038/s41598-017-15520-4)
Supplement: Supplementary file 1 — Supplementary Information [file 41598_2017_15520_MOESM1_ESM.pdf]

Supplementary Information

The photocycle of orange carotenoid protein conceals distinct intermediates and asynchronous changes in the carotenoid and protein components

E.G. Maksimov, N.N. Sluchanko, Y.B. Slonimskiy, E.A. Slutskaya, A.V. Stepanov, A.M. Argentova-Stevens, E.A. Shirshin, G.V. Tsoraev, K.E. Klementiev, O.V. Slatinskaya, E.P. Lukashev, T. Friedrich, V.Z. Paschenko, A.B. Rubin

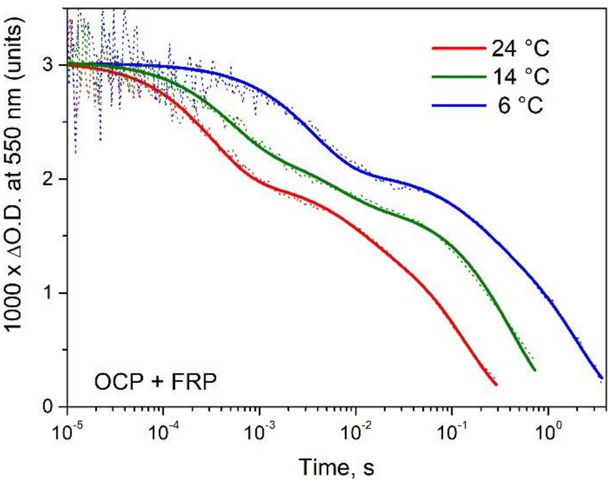

Figure S1. Characteristic flash-induced transitions of WT OCP absorption at 550 nm at different temperatures approximated by multiexponential decay. All samples contained OCP and FRP (1/1.6 concentration ratio).

The absence or low efficiency of excitation energy transfer (EET) in the red state could be at least partially explained by the changes of overlap integral between the Trp emission (donor) and ECN absorption (acceptor), which decreases due to the red shift of ECN absorption in the photoactivated state. Another reason could be the increased mobility of carotenoid molecule in the red state, which may lead to reduction of the role of (specific) orientation factors between Trp residues and the carotenoid. It should be noted that at least at low temperatures, the average Trp fluorescence lifetimes of OCP^R are significantly larger compared to that of the OCP apoprotein (Apo-OCP) (*Supplementary Figure 2*), indicating that in this state quenching is not only removed, but that the carotenoid provides a hydrophobic environment for some Trp residues, which would shield them from collisional interactions with water compared to the situation in Apo-OCP. Previously it was shown that the largest solvent accessibility changes after photoconversion occurred in CAN binding residues, namely a decrease in solvent accessibility of Trp-41 [26].

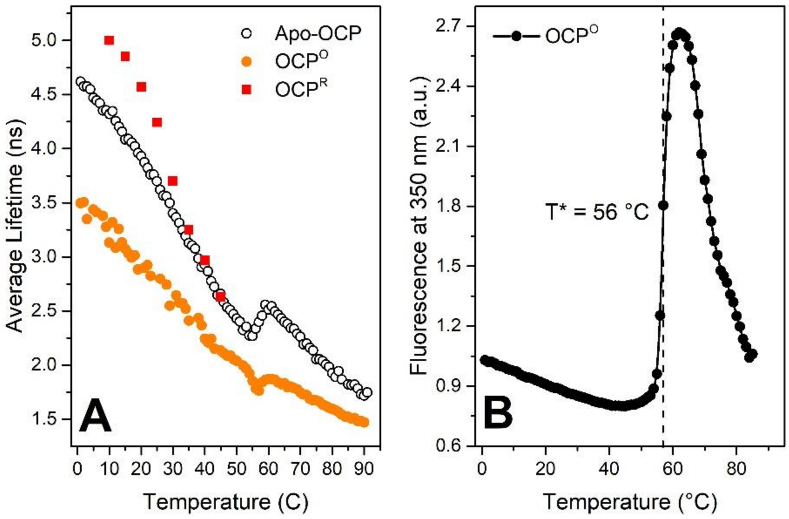

Figure S2. (**A**) - temperature dependency of tryptophan average fluorescence lifetime of OCP^O, OCP^R and Apo-OCP registered at 350 nm. (**B**) – temperature dependency of fluorescence intensity of OCP^O T* indicates half-transition (melting) temperature.

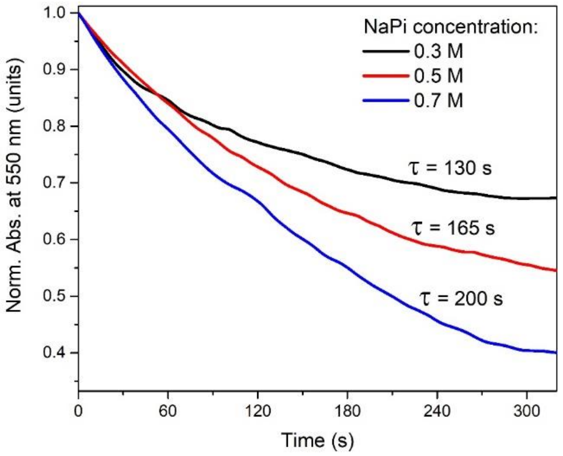

Figure S3. Time-courses of red to orange back-conversion recorded as absorption changes at 550 nm of initially non-photoactive purple OCP-W288A mutant in presence of different concentrations of phosphate.

The Stern-Volmer plots of Apo-OCP and OCP^R also exhibited some slight negative deviation from linearity at iodide concentrations above 500 mM, which on the first sight also suggests limited accessibility of Trp residues; however, the modified Stern-Volmer plots for these species (*Figure 4F*) yield an accessible fraction (f_a) of 1. This indicates that some other effect limits the quenching at high concentrations of iodide. Since iodide anions belong to the group of chaotropes in the Hofmeister series, it is possible that high iodide concentrations induce slight (local) destabilizations of the protein structure, which could entail changes in the microenvironment of one or the other Trp residue.

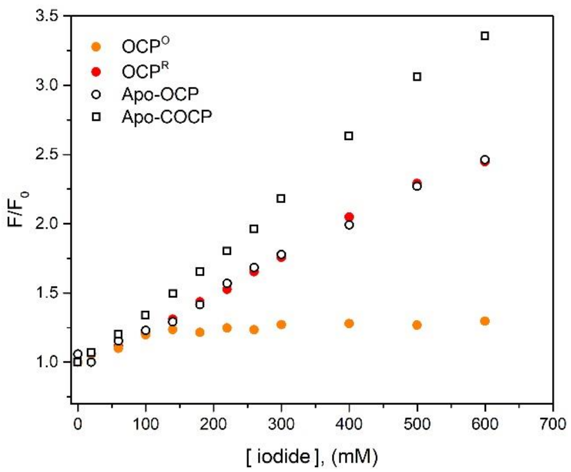

Figure S4. Stern-Volmer plots for Apo-COCP, Apo-OCP, OCP^O and OCP^R.
